# Economic impact of gastrointestinal nematodes in Morada Nova sheep in Brazil

**DOI:** 10.1590/S1984-29612022044

**Published:** 2022-08-19

**Authors:** Ana Carolina de Souza Chagas, Oscar Tupy, Isabella Barbosa dos Santos, Sérgio Novita Esteves

**Affiliations:** 1 Embrapa Pecuária Sudeste – CPPSE, São Carlos, SP, Brasil; 2 Faculdade de Ciências Agrárias e Veterinárias – FCAV, Universidade Estadual Paulista – UNESP, Jaboticabal, SP, Brasil

**Keywords:** Production loss, weight impact, death rate, Haemonchus contortus, small ruminants, anthelmintic resistance, Perda de produção, impacto no peso, taxa de mortalidade, Haemonchus contortus, pequenos ruminantes, resistência anti-helmíntica

## Abstract

This study evaluated the economic impact of gastrointestinal nematode (GIN) infection in Morada Nova lambs under different parasite chemical control conditions. For this, 246 lambs, in the rainy and dry season, were randomized into groups according to their anthelmintic treatment with levamisole: control (CT: no treatment); routine treatment (RT: treated every 42 days); and targeted selective treatment (TST: treated according to the average daily weight gain, DWG). From 63 days of age (D63) to D210, the lambs were weighed and monitored for GIN infection parameters. Spending on anthelmintics in the production system was 1.3% of the total economic result. The economic result per animal (R$ 5.00 = US$ 1.00) was higher in the RT group, amounting to US$ 6.60 in the rainy and US$ 5.69 in the dry season, due to higher DWG. Thus, RT presented economic results 14.4% and 10.9% higher than CT, and 7.2% and 1.9% higher than TST, in the rainy and dry season, respectively. However, fast development of resistance made RT unfeasible. Here, the economic impact of GIN infection on a national scale is discussed, demonstrating its importance and the impossibility of profitable and sustainable sheep production without adequate control.

## Resumo

Este estudo avaliou o impacto econômico da infecção por nematoides gastrintestinais (NGI), em cordeiros Morada Nova, sob diferentes condições de controle químico dos parasitas. Para isso, 246 cordeiros, na estação chuvosa e seca, foram randomizados em grupos de acordo com o tratamento com levamisol: controle (TC: sem tratamento); tratamento rotineiro (TR: tratado a cada 42 dias); e tratamento seletivo direcionado (TST: tratado de acordo com o ganho de peso médio diário, GMD). Dos 63 dias de idade (D63) ao D210, os cordeiros foram pesados ​​e monitorados quanto aos parâmetros de infecção por NGI. O gasto com anti-helmínticos no sistema produtivo foi de 1,3% do resultado econômico total. O resultado econômico por animal (R$ 5,00 = US$ 1,00) foi maior no grupo RT, totalizando US$ 6,60 na estação chuvosa e US$ 5,69 na seca, devido ao maior GMD. Assim, o RT apresentou resultados econômicos 14,4% e 10,9% superiores ao TC, e 7,2% e 1,9% superiores ao TST, no período chuvoso e seco, respectivamente. Entretanto o rápido desenvolvimento de resistência inviabiliza o TR. O impacto econômico da infecção por NGI em escala nacional são aqui discutidos, demonstrando sua importância e a impossibilidade de uma ovinocultura lucrativa e sustentável sem o controle adequado.

## Introduction

Brazil has 19.7 million sheep, and the national flock increased by 4.05% in 2018 (IBGE, 2019). The northeastern region has 13.5 million head, equivalent to 71.05% of the national flock, followed by the southern, central-western and southeastern regions, with 3.95 million, 1.0 million and 600,000 head, respectively, corresponding to 20.53%, 5.26% and 3.16% of the total Brazilian sheep flock (IBGE, 2019; Magalhães et al., 2020). However, due to lack of organization in the production chain, sheep production still does not supply the domestic market with efficiency and quality. One of the biggest problems relates to the deficit in constancy of supply, which makes it difficult to structure the sector, including the setting of slaughter scales. Consequently, importing of sheep meat becomes necessary: in 2020, about 3,219 tons were imported (Zen et al., 2014; FAO, 2020).

Parasitic diseases caused by gastrointestinal nematodes (GIN) are among the factors that limit the production of small ruminants worldwide. They are responsible for high economic losses, due to slow growth, weight loss, reduced food consumption, reduced milk production, diminished fertility and, in cases of massive infection, high mortality rates. These clinical signs are caused by lesions in the gastrointestinal mucosa, which disturb nutrient absorption; and by hematophagous spoliation, which leads to subclinical infection. In some cases, these effects are associated with reduced weight gain and anemia and, consequently, with low body condition and carcass yield. *Haemonchus contortus* is the most clinically and economically important nematode in small ruminant farming in Brazil (Cavalcante et al., 2009; Chagas et al., 2013). Females can release between 5,000 and 10,000 eggs daily (Romero & Boero, 2001). Their pre-patent period, i.e. the period between ingestion of the infective larvae (L_3_) by the host and elimination of eggs in feces, is 18 to 22 days (Santos et al., 2014). *H. contortus* settles in the abomasum and can ingest 0.05 to 0.08 mL of blood/day. Therefore, when animals have high parasite loads, they present anemia and submandibular edema and may die (Amarante, 2014).

GIN control has been largely carried out through use of anthelmintics. However, excessive dependence on these substances has led to the development of anthelmintic resistance (Muchiut et al., 2018; George et al., 2021; Viana et al., 2021). It is important to understand the extrinsic factors that favor the development of anthelmintic resistance, as these have a direct impact on flock productivity (Waller, 2006). In this way, the development of modeling approaches should allow scaling-up from studies concerning expenses for ineffective anthelmintics, to enable predictions about farmers’ profits.

In addition to the worldwide resistance, studies of the potential environmental consequences of excessive anthelmintic administration to sheep indicate that this approach is not sustainable, so its impact should be considered. The period of maximum residue excretion is generally more transient in sheep than cattle dung, but low-level excretion may continue for longer, giving the potential for extended sub-lethal effects (Beynon, 2012). This has a huge impact on *refugia* in pastures and on the future efficacy of the chemical groups in flocks. According to Boxall et al. (2007), anthelmintics administered to sheep enter into the environment more through feces than urine, but the wash-off of topically applied compounds, spillage during application and inappropriate disposal of compounds also provide other important environmental entry points. Thus, beyond the negative impact on non-target species in soil and dung, contamination also reaches other ecosystems, such as in groundwater, surface water bodies and watercourses. Since the complexity of the drug-dung-fauna system is challenging to observe and quantify *in vivo* and is difficult to fully represent under controlled laboratory conditions, modeling techniques are also alternatives to address these issues (Cooke et al., 2017).

Concerning the economic impact, the annual costs for GIN control among small ruminants outweigh all other costs for endemic disease control in developed countries. In the three largest sheep producing countries (Australia, South Africa and Uruguay), losses due to helminth infection are around US$ 222 million, US$ 45 million and US$ 42 million, respectively (Waller, 2006). In Brazil, on the other hand, studies of the economic impact of GIN infection in ruminants are more abundant in relation to cattle, mainly regarding reduced weight gain (Bianchin et al., 1995; Grisi et al., 2014; Heckler et al., 2016; Oliveira et al., 2021). In sheep, a survey of parasitic diseases diagnosed in animals in a region of the state of Rio Grande do Sul, Brazil, indicated that 42.7% of the cases diagnosed consisted of GIN infection. These gave rise to mortality of approximately 16,800 animals per year, which resulted in an economic impact estimated at US$ 400,000 (Oliveira et al., 2017). Thus, the objective of this study was to evaluate the economic impact of GIN infection among Morada Nova lambs under different conditions of parasite chemical control.

## Material and Methods

### Experimental groups

This experiment were approved by the local Ethic Committee on Animal Experimentation (process no. CEUA 01/2020), and are in accordance with national and international ethical principles and guidelines for animal experimentation.

During the rainy season, ewes were fed exclusively on pasture, and in the dry season, they received corn silage supplementation (gradually increasing as the pasture supply decreased) and feed concentrate at a proportion of 1% of the live weight. Water and mineral salt were kept available *ad libitum*. Their lambs, of both sexes, were reared together with the dams until the mean age of weaning, at 150 days of age, when the ewes were removed from the pasture area. The lambs then remained on the pasture until the end of the experiment at 210 days of age (D210), on average.

Thus, 144 and 102 Morada Nova lambs were evaluated in the rainy season of 2019 and dry season of 2020, respectively. At 63 days of age (D63), all the animals received anthelmintic treatment (Ripercol® L - 150F; injectable levamisole 18.8%, 9.4 mg/kg), to start the comparative experimental treatments. From D63 to D210, every 21 days, blood and feces samples were collected for hematocrit measurement (packed cell volume, PCV), individual eggs per gram of feces (EPG) counts (Ueno & Gonçalves, 1998) and fecal culture per group (Roberts & O'Sullivan, 1950). All the animals were weighed every 21 days from birth to D210.

On D63 of each season, the lambs were then divided into three groups (with homogeneous means for birth weight, type of birth (single or twin), sex, EPG and PVC), according to the anthelmintic treatment (injectable levamisole 18.8%, 9.4 mg/kg) proposed: control (CT): no treatment; routine treatment (RT): treatment of all lambs every 42 days, from D105 to D189; and targeted selective treatment (TST): treated when the average daily weight gain of the lamb (DWG) was ≤ mean DWG of the TST group - (standard deviation of the TST group’s DWG * 0.5) (adapted from Cintra et al., 2018), every 21 days, from D105 to D189. Lambs that presented PCV ≤ 21% received anthelmintic treatment and the most debilitated animals were supplemented with vitamin B12 in order to accelerate recovery and avoid deaths. B12 administration occurred only in CT.

The average cost of time spent on labor, to feed animals, collect feces, make blood and weight measurements and do other activities, was considered similar in the three treatments during the experimental period. The cost of handling the herd was estimated at US$ 0.10/animal/day.

### Economic result

The economic analysis (R$ 5.00 = US$ 1.00) was conducted after obtaining data from the lambs born and weaned in the rainy season of 2019 and in the dry season of 2020, when all parameters had been measured and collected. The experimental treatments were then compared regarding their economic results, which were obtained through Equations 1, 2 and 3. The gross economic result was the variable used for comparison.


GR = NA x W x US$/kg BW (1)

GE = GR – OPC (2)

$$OPC\;=\;\sum \left(ahC;\;vitC;\;supC;\;lhC\right)$$ (3)

The gross revenue from the sale of animals (GR) was obtained through Equation 1: number of animals per treatment (NA) x average weight at 210 days (W) x market price paid in dollars (US$/kg of body weight). The gross economic result (GE) was obtained through Equation 2: GR – operating cost per treatment (OPC). The GE per animal was obtained by dividing the GE by the number of animals per treatment. The OPC (Equation 3) was obtained as the sum of the operational cost per treatment (anthelmintic – ahC; vitamin B12 – vitC; supplies such as syringes, needles etc. – supC; and labor for handling herd – lhC). Pasture maintenance costs, food supplements, depreciation and land costs were not considered, as these remained constant for all treatments.

## Results

The results regarding GIN infection (PCV, EPG and fecal cultures) and its impact on weight gain are described more fully in a study of the consequences of different anthelmintic treatments (CT, RT and TST) on parasite control in the rainy and dry season (Santos et al., 2022). The main results are summarized in Table 1. There was a statistical difference in the overall mean EPG between CT and RT in the rainy season and among the three treatment groups in the dry season. Regarding the PCV, RT was statistically similar to TST only in the dry season. On D210, the mean weight in RT exceeded those of the other treatments just in the rainy season. RT presented statistically higher mean DWG than the other treatments in the rainy season, but it was not different from TST in the dry season. In comparison with RT (100% anthelmintic treatment), the TST presented an average percentage of 27.2% of lambs dewormed in the rainy season and 32.8% in the dry season, while the CT presented 10.5% and 4.4%, respectively, when PCV ≤ 21%. Levamisole had the lowest efficacy in the FECRT and the highest LCs in the RESISTA-Test© for RT, in both seasons. The PCR indicated that the polymorphism was under selective pressure (p ≤ 0.05) on D210 for all treatments and seasons.

**Table 1:** Means of eggs per gram of feces (EPG) counts, packed cell volume (PCV), average live weight on D210 (LW), daily weight gain (DWG), and number (percentage) of anthelmintic treatments in control (CT), routine (RT) and targeted selective (TST) treatments, in Morada Nova lambs, during the rainy (R) and dry (D) seasons (S). Also data concerning resistance to levamisole on D210: lethal concentrations (LC_50_, µg.mL^-1^) obtained by the RESISTA-Test©, percentages of anthelmintic efficacy in FECRT and percentages of *Haemonchus contortus* with resistant genotypes by PCR.

**S**	**Treat**	**EPG**	**PCV**	**LW**	**DWG** ^*^	**N°**	**LC_50_**	**FECRT** ^**^	**PCR** ^***^
**R**	**CT**	4665.1a	32.1b	20.94b	0.087b	12 (10.5%)	0.482	66.4	57.1
	**RT**	3063.5b	33.4a	23.92a	0.101a	114 (100%)	1.926	24.1	71.4
	**TST**	3462.0ab	32.3b	22.28b	0.094b	62 (27.2%)	0.117	76.4	40.0
**D**	**CT**	4475.1a	33.9b	24.86a	0.102b	6 (4.4%)	0.437	90.7	47.8
	**RT**	1341.7c	36.0a	26.59a	0.113a	102 (100%)	0.851	12.4	55.9
	**TST**	2863.4b	35.1a	25.90a	0.112a	67 (32.8%)	0.045	64.8	41.9

Different letters in the same column indicate statistical difference between treatments by Tukey test (p ≤ 0.05), within each season. More detailed data are shown in Santos et al. (2022).

* DWG calculated from D21 to D210.

** FECRT = Fecal egg count reduction test;

*** PCR = Polymerase chain reaction test.

In the present study, the RT animals reached D210 heavier (23.92 kg) than the TST (22.28 kg) and CT (20.94 kg), thus generating higher gross revenue (US$ 1,946.92) than the TST (US$ 1,813.42) and CT (US$ 1,750.76), in the rainy season. This difference in weight gain is believed to have been triggered by anthelmintic treatments, which were more frequent in RT than in TST or CT, as mentioned above. The same pattern was observed in the dry season for RT (US$ 1,995.80), TST (US$ 1,899.79) and CT (US$ 1,798.94) (Table 2).

**Table 2:** Economic result regarding GIN infection control in the control (CT), routine (RT) and targeted selective (TST) treatments, among Morada Nova lambs in the rainy and dry seasons.

**Season**	**Rainy**			**Dry**		
**Variables**	**CT**	**RT**	**TST**	**CT**	**RT**	**TST**
**Number of animals per treatment**	38	38	38	34	34	34
**Initial live weight at 63 days**	11.23	11.42	11.51	10.54	10.14	10.50
**Final live weight at 210 days**	20.94	23.92	22.28	24.86	26.59	25.90
**Average daily gain in grams** ^*^	66	85	73	97	112	105
**Gross revenue (sale of animals - US$)**	1,750.76	1,946.92	1,813.42	1,798.94	1,995.80	1,899.79
**Total cost of deworming (US$)**	26.52	25.72	21.88	21.46	24.83	22.08
**Total economic result (US$)**	1,724.24	1,921.20	1,791.54	1,777.48	1,970.97	1,877.71
**Economic result/head (US$)**	45.32	51.92	48.42	52.28	57.97	56.90
**Price received per kg of live weight (US$)**	2.20	2.20	2.20	2.20	2.20	2.20

* DWG calculated from D63 to D210.

During the rainy season there were 12 anthelmintic treatments in the CT, 62 in the TST and 114 in the RT (38 animals x 3 treatments). In the dry season, there were 6, 67 and 102 treatments (34 animals x 3 treatments), respectively. From these data, the total expenditure on anthelmintic treatments per experimental group and per season was calculated (Table 2). The total cost of anthelmintic application, considering materials and labor, was US$ 17.88, US$ 25.72 and US$ 21.88 for the CT, RT and TST treatments, respectively, in the rainy season. CT had lower expenditure on application of anthelmintic due to the small number of animals (n = 12) that presented PCV ≤ 21%. However, due to vitamin B12 supplementation for debilitated animals, the total cost of the CT increased by US$ 8.64, to total US$ 26.52, which was higher than the cost of the other treatments. Spending on anthelmintics represented 1.5%, 1.3% and 1.2% of the total economic results for CT, RT and TST, respectively, in the rainy season. The RT treatment consumed a greater amount of anthelmintics, syringes and needles, which were applied every 42 days, but despite this, it presented an economic gain 14.4% higher than the CT and 7.2% higher than the TST. Due to the higher DWG obtained between D63 and D210 for this group, the economic gain per animal was US$ 51.92, while for the TST and CT it was US$ 48.42 and US$ 45.32, respectively (Table 2).

In the dry season, the total costs of anthelmintic application were US$ 17.14, US$ 24.83 and US$ 22.08 for the CT, RT and TST treatments, respectively. The CT had the lowest cost, as only six animals had PCV ≤ 21%. However, with vitamin B12 supplementation, US$ 4.32 was added, to total US$ 21.46, which was lower than the cost of the other treatments. The total expenditures on anthelmintics represented 1.2%, 1.3% and 1.2% of the total economic result for CT, RT and TST, respectively. Even with the lower cost, the CT showed a lower economic result, due to the lower weight gain, while the RT was 10.9% higher than the CT and 1.9% higher than the TST. The economic results per animal for the RT, TST and CT were US$ 57.97, US$ 56.90 and US$ 52.28, respectively (Table 2).

Table 3 shows the net differences in economic result per head between the treatments for the two experimental periods. In the rainy season, the economic result per head was significantly better in the RT, by US$ 6.60/animal, compared with the CT; and by US$ 3.48, compared with the TST. In the dry season, the result for the RT was better than for the CT, by US$ 5.69. However, in relation to the TST, the result for the RT was better only by US$ 1.07, a much smaller difference than what was observed in the rainy season.

**Table 3:** Differences in economic result (US$) per head between the control (CT), routine (RT) and selective (TST) treatments for Morada Nova lambs in the rainy and dry seasons.

**Season**	**Treatment**	**CT**	**RT**	**TST**
	CT	0.00	-6.60	-3.10
**Rainy**	RT	6.60	0.00	3.48
	TST	3.10	-3.48	0.00
	CT	0.00	-5.69	-4.62
**Dry**	RT	5.69	0.00	1.07
	TST	4.62	-1.07	0.00

## Discussion

The most intense treatment of the entire flock with anthelmintic, done every 42 days (RT), proved to be the most interesting one in economic terms. In general, this strategy made it possible to obtain a higher DWG among the animals, while favoring a lower level of GIN infection (EPG) and positively impacting the weight performance of the animals. The present study also indicated that when farmers use anthelmintic treatment only when the animal is already in an intensely anemic state, this may, at first sight, seem to be less expensive. Nevertheless, the potential for deaths or for expenditure on vitamins or other support medicines contradicts this hypothesis.

On the other hand, although the cost of intense anthelmintic treatment was relatively higher (since levamisole is inexpensive), a relatively higher final live weight of animals was obtained in the RT, such that better economic results per head were attained under these experimental conditions, mainly in the rainy season. However, studies carried out in Brazil have shown that anthelmintic resistance develops quickly under conditions of intense and non-selective parasite control. This type of management (RT) should not be implemented as a practice in farms, as it will make sustainability of the production chain unfeasible. Parasites from sheep intensely treated with monepantel in Brazil showed resistance to this drug within three months (Albuquerque et al., 2017). Faster establishment of resistance was also detected among Morada Nova lambs in the state of São Paulo after the third treatment with levamisole, performed every 42 days (dos Santos et al., 2022).

Conversely, the TST approach allowed good weight performance, very close to the RT, but promoted reduction in the use of anthelmintics. The number of treatments in TST was almost half of the number in RT, and on average, 30% of the TST lambs received anthelmintics. Studies have shown that when TST is adopted, the interval between anthelmintic use and development of resistance is longer. This also results in less impact on the environment and less drug residues in animal products (dos Santos et al., 2022). In flocks in which TST was performed using EPG as a parameter, it took around 4.5 years for anthelmintic resistance to ivermectin to become established (Echevarria & Trindade, 1989). On farms in the southern region of Brazil on which lambs received less than three treatments of different classes of anthelmintics per year, the anthelmintic resistance rate detected was 6.7%; while when they received four to six treatments per year, it was 44.9%; and when they received seven or more treatments, it was 48.3% (Echevarria et al., 1996). In the Netherlands, the first report of resistance to monepantel occurred on a farm where this drug was administered more carefully for two years, twice a year for ewes and sires, and on average three times a year for lambs (van den Brom et al., 2015).

Anthelmintics have been the main tool adopted for parasite controls and have usually positively impacted the welfare and health of domestic and production animals (Pasiani et al., 2012). However, in a scenario of highly resistant parasites (Raschia et al., 2021), *refugia* preservation, favored by the TST approach, has become essential for maintaining the efficacy of anthelmintics, which must be used carefully (George et al., 2021). As highlighted by Sauermann et al. (2020), any parasite in a pasture is only in *refugia* if it successfully develops to the adult stage and produces viable offspring. Otherwise, it does not contribute to the population genetics and must be disregarded in terms of *refugia*. Thus, there is a tendency to a shift away from whole-flock treatments to the TST approach, which will decrease the negative impacts of treatments on dung fauna populations by providing population *refugia*. This provides novel evidence for the benefits of TST regimens to local food webs (Cooke et al., 2017). Another point concerning the overuse of anthelmintics due to resistance is the presence of residues in food and in the environment. The rise in global temperature is leading to increased occurrence and alterations in the distribution of many infectious diseases. Recent reports by the Intergovernmental Panel on Climate Change and the Food and Agriculture Organization of the United Nations identified an increase in livestock diseases, including parasite infections, as a result of changing climate, with a negative impact on food security (Sauermann et al., 2020).

A study carried out in in the state of Ceará, Brazil, indicated that helminthiasis accounted for 81.9% of the diseases diagnosed in goats and sheep (Pinheiro et al., 2002). In the state of Paraná, a study on postmortem diagnoses among 177 sheep showed that haemonchosis was the main disease diagnosed, affecting 53 (20.87%) animals (Sprenger et al., 2015). In the central region of Rio Grande do Sul, it was found that parasitosis accounted for 24.3% of all diagnoses and that 62.5% of these parasitoses consisted of haemonchosis (Rissi et al., 2010). In a survey of the most frequent parasitic diseases in sheep in the southern region of Rio Grande do Sul, covering the period from 1978 to 2014, Oliveira et al. (2017) found that 33.6% of the diagnoses made were of parasitic infections and that mixed gastrointestinal parasites (42.7%) and haemonchosis (35.4%) together accounted for 78.1% of the diagnoses among sheep of all ages. Based on the estimated mortality rate of 5% for the species (Rio Grande do Sul, 2010) and the percentage of sheep diagnosed with parasitic infection (33.6%), the estimated annual economic losses were US$ 400,000 in the flocks of the southern region of Rio Grande do Sul alone, which total around one million sheep (Oliveira et al., 2017). Indirect losses such as the decrease in production (meat, milk and wool) and expenditure on medicines and veterinary care were not considered.

Based on the aforementioned data, it was then possible to estimate the losses due to parasitic infections in all Brazilian flocks (Table 4), considering an average percentage parasitic morbidity of 30.0% (range of values between 24.3% and 33.6%), with 5.0% mortality and 78.1% parasitosis, which have been observed in sheep flocks (Rissi et al., 2010; Oliveira et al., 2017). The current average value of US$ 44.00 per lamb was also considered. Thus, we calculated that these assumed values added up to estimated total losses of US$ 107.52 million per year. These losses may be even higher if the expenditure on medicines and veterinary assistance for prevention of parasitic diseases are added (Table 4). From the data of the present study, if we consider a national mortality rate of 295,500 sheep per year due to parasitic diseases, the losses due to deaths in the Brazilian sheep industry can be estimated as US$ 13 million per year. The biggest losses are caused by the reduction in weight gain and these reach US$ 94.5 million per year (Table 4). This was obtained by multiplying the number of animals affected by parasitic diseases by the average value (US$ 6.14) of the difference in the gain through the TST in relation to CT, between the rainy season (US$ 6.60) and dry season (US$ 5.69) (Table 3).

**Table 4:** Estimated number of sheep in Brazil in 2019 (IBGE, 2019) and estimated losses due to lower weight gain and death caused by gastrointestinal nematodes per year and according to region.

**Region**	**Number of sheep**	^*^ **Estimate of the number of sheep killed by parasites**		^**^ **Estimate of the number of sheep with reduced weight gain**		^***^ **Losses due to reduced weight gain (US$)**		^****^ **Losses through deaths caused by parasites (US$)**		**Total per year (US$)**
**North**	600,000	9,000	468,600		2,879,078.40		396,000.00		3,275,078.40	
**Northeast**	13,500,000	202,500	10,543,500		64,779,264.00		8,910,000.00		73,689,264.00	
**Southeast**	600,000	9,000	468,600		2,879,078.40		396,000.00		3,275,078.40	
**South**	4,000,000	60,000	3,124,000		19,193,856.00		2,640,000.00		21,833,856.00	
**Midwest**	1,000,000	15,000	781.000		4,798,464.00		660,000.00		5,458,464.00	
**Total**	19,700,000	295,500	15,385,700		94,529,740.80		13,002,000.00		107,531,740.80	

* considering that 30% of disease diagnoses in sheep are parasitic diseases and that the mortality rate is 5%;

** considering that the parasite occurrence rate in the flock is 78.1%;

*** estimated loss of US$ 6.14 per animal per year;

**** considering US$ 44.00 per animal.

Given that information on production losses in economic studies is usually obtained from control animals (which are kept untreated or do not have GIN infection), estimates of economic losses represent potential losses expected in the absence of parasite control measures (Grisi et al., 2014). On the other hand, considering that the current scenario of parasite resistance results in frequent anthelmintic treatment, generally using commercial products without efficacy against GIN, this increases the damage to production systems by including the costs of ineffective control. Therefore, a need exists for further studies to evaluate the contribution of anthelmintic resistance to the economic damage resulting from parasitic diseases and the impact of anthelmintics on the environment and human health.

The reduction in weight gain is a consequence of parasitism, as also are occurrences of deaths. The latter was avoided in the present study due to animal welfare issues, but it represents 12.1% (US$ 13 million) of the total annual losses caused in sheep farming. The most significant losses for Brazil occur in the northeastern region, which has the largest number of small ruminants. Regarding weight loss, a study on Dorper lambs in Brazil was carried out with the following groups: infected-supplemented (G1), control-supplemented (G2), infected-basal diet (G3) and control-basal diet (G4). The control groups received anthelmintic treatment every 14 days to minimize GIN infection. The lambs that received anthelmintic treatment (G2 and G4) showed 17.1% and 26.7% greater daily weight gain, respectively. For the supplemented lambs, the difference was smaller, as their control group was also supplemented (Starling et al., 2019). The data from the present study showed that the final weight (D210), in relation to the initial weight (D63), was 28.8% and 15.5% higher in the rainy and dry seasons, respectively. These percentages were close to those of the above study. In addition, the present study, carried out for 147 days, showed DWGs that were much lower than those of Starling et al. (2019), thus indicating the relevance of breed and food supplementation level in economic estimates. As is well known, supply of adequate food according to animal category, especially in relation to crude protein, is of great importance for parasitism control and for enabling less need for anthelmintic treatment, thereby contributing to the sustainability of the chain (Bricarello et al., 2005; Cériac et al., 2019).

The values estimated here would need to be better evaluated at the regional level. Parasite occurrence depends, for example, on elements such as: temperature, rainfall, soil, topography, pasture type and management, species, breed, age, physiological and nutritional status and animal management (Ruas & Berne, 2001). Morada Nova lambs are resilient to GIN and adults are resistant (Issakowicz et al., 2016; Toscano et al., 2019; Haehling et al., 2021; Okino et al., 2021). Thus, we believe that the values calculated here are underestimates in relation to more susceptible breeds, such as Suffolk and Ile de France (Amarante et al., 2004). In addition, the anthelmintic adopted here has a lower cost than monepantel-based products, for example. The data from the literature was reliable for our calculations. Thus, we expect this model framework can be adapted to any system, anywhere, given workable parameter estimates. Data can be adjusted with local information for the variables in Table 2, which will feed the regional estimates presented in Table 4.

Regardless of the limitations of studies that are used to develop economic estimates, especially when extrapolated from local situations to a national scale, the picture obtained here demonstrates the magnitude and importance of parasitism in Brazil and the impossibility of profitable livestock-rearing without adequate control of GIN (Grisi et al. al., 2014). In addition, fast establishment of anthelmintic resistance, which currently occurs in the majority of the national flocks, has an important impact that is difficult to incorporate into the calculation of the economic damage resulting from parasitic diseases. Associated with that, there is a lack of knowledge or understanding of the functional consequences of eco-toxic residues’ effects. Thus, an integrated approach between ecologists and economists should be further explored, in order to increase understanding of the economic importance of maintaining functional pasture systems, by including an economic variable to ecosystem functions (Beynon, 2012). Future studies should also include economic analysis, in order to balance short-term production gains with longer term environmental impacts (Cooke et al., 2017).

## Conclusion

The animals in the RT reached 210 days of age heavier than those in the CT, thus generating higher gross revenue in both seasons. In relation to the CT, it was possible to verify that use of RT provided a significantly better economic result, in both seasons. The expenditure on use of anthelmintics had low impact. However, under conditions of intense use of anthelmintics (i.e. in the RT group), resistance is quickly established in parasites, thus making the production system unfeasible. The TST approach has potential long-term economic benefits and can play important role in reducing environmental impacts. The panorama here discussed demonstrates the importance of parasitism by GIN in Morada Nova flocks and the impossibility of profitable livestock-rearing without rational control of parasites that include approaches aiming to delay the development of anthelmintic resistance.
